# Electrical Transition in Isostructural VO_2_ Thin-Film Heterostructures

**DOI:** 10.1038/s41598-019-39529-z

**Published:** 2019-02-28

**Authors:** Adele Moatti, Ritesh Sachan, Valentino R Cooper, Jagdish Narayan

**Affiliations:** 1 Materials Science and Engineering, North Carolina State University, Raleigh, NC 27606 USA; 2 Materials Science Division, Army Research Office, Research Triangle Park, Raleigh, NC 27709 USA; 3 Materials Science and Technology Division, Oak Ridge National Laboratory, Oak Ridge, TN 37830 USA

## Abstract

Control over the concurrent occurrence of structural (monoclinic to tetragonal) and electrical (insulator to the conductor) transitions presents a formidable challenge for VO_2_-based thin film devices. Speed, lifetime, and reliability of these devices can be significantly improved by utilizing solely electrical transition while eliminating structural transition. We design a novel strain-stabilized isostructural VO_2_ epitaxial thin-film system where the electrical transition occurs without any observable structural transition. The thin-film heterostructures with a completely relaxed NiO buffer layer have been synthesized allowing complete control over strains in VO_2_ films. The strain trapping in VO_2_ thin films occurs below a critical thickness by arresting the formation of misfit dislocations. We discover the structural pinning of the monoclinic phase in (10 ± 1 nm) epitaxial VO_2_ films due to bandgap changes throughout the whole temperature regime as the insulator-to-metal transition occurs. Using density functional theory, we calculate that the strain in monoclinic structure reduces the difference between long and short V-V bond-lengths (Δ_*V*−*V*_) in monoclinic structures which leads to a systematic decrease in the electronic bandgap of VO_2_. This decrease in bandgap is additionally attributed to ferromagnetic ordering in the monoclinic phase to facilitate a Mott insulator without going through the structural transition.

## Introduction

The metal-insulator transition in strongly correlated materials such as vanadium dioxide (VO_2_) is usually coupled with the symmetry-lowering structural transition, which is tetragonal rutile P 4_2_/mnm to monoclinic P 2_1_/c. The fundamental understanding and control over electrical and structural transitions in VO_2_, which occur often simultaneously, are of immense scientific importance with profound impact on technological applications ranging from smart switching to infrared sensing devices. Over the years, numerous efforts have been made in this direction, primarily focusing on the manipulation of these transitions via defect and interface engineering^1–5^. However, the switching speed and endurance of such devices are often limited by the complexities that emerge from the kinetically slower occurrence of the structural transition (10 picoseconds) as compared to the electrical transition (0.1 picoseconds)^6–8^. This leads to the decoupling between these coexisting transitions in the presence of strain, dopants, and defects in the thermal spectrum and deleteriously affects the performance of such systems^2,4,8,9^. The coexistence of electrical and structural transitions presents practical challenges in fabricating electronically-correlated VO_2_ based solid-state devices^3^. In this respect, the development of materials displaying an isolated electrical transition without an accompanying structural transition provides an ideal solution. This can be achieved by strain management in VO_2_ thin films^10–13^. It has been shown that the primary mechanisms of metal-insulator transitions are based on electron-electron interactions (Mott transition) and electron-lattice interactions (Peierls transition). The ratio of these can be effectively steered through strain-induced tuning of c/a lattice ratio in VO_2_ thin films^1,14–18^. This is a result of an interplay between these competing mechanisms of electron-electron interaction and electron-phonon interaction, leading to a tunable electrical transition^1,12,15,19^. Previously, several researchers including our group have shown that it is possible to separate structural and electrical transitions^9,10,20–23^. However, this raises the question of whether it is possible to totally prevent the occurrence of the structural transition, which had been predicted previously by density functional theory (DFT) calculation suggesting a thermally stable monoclinic metallic phase of VO_2_^24^. The insulating state in monoclinic VO_2_ results from electron-electron correlations and electron-phonon interactions. These correlations can be manipulated by charge, spin, orbital, and lattice degrees of freedom. This means that the ratio of the Mott (electron-electron correlations) and Peierls (electron-phonon interactions) transitions can change depending on these factors. Thus, if one of these factors can be pinned forcibly in VO_2_, the correlation is modified accordingly and an unusual behavior might be realized. Recently, some efforts have been devoted to the suppression of the temperature-dependent structural phase transition in VO_2_^10,14^.

In our work, we have designed a unique non-equilibrium isostructural monoclinic (no temperature dependency) VO_2_ phase on a practical substrate, which demonstrates an uninterrupted insulator-metal transition without undergoing a structural change. This pseudomorphic structure is compared with a fully relaxed VO_2_ thin film grown on the same heterostructure above the critical thickness. This unique integration allows us to observe both strain-trapped and fully relaxed VO_2_ thin films behavior in the same heterostructure. Basically, we have employed trapped misfit strain to pin the lattice degree of freedom, while leaving the charge-orbital interactions free to transform at the transition temperature. We expect that the structure pinning in the isostructural VO_2_ to affect orbital configuration and bandgap, leading only to the electrical transition with temperature. We have monitored both transitions using *in-situ* electrical and structural characterization techniques. This phenomenon has a profound impact from an application standpoint since the volume changes of ~0.32% across the structural transition in conventional VO_2_ films can cause mechanical instability, microcracking, and deterioration in electrical and optical properties^25^. Besides, the switching speed can also be enhanced since the kinetically slower occurrence of the structural transition (10 picoseconds) as compared to the electrical transition (0.1 picoseconds) has been eliminated. The demonstration of structurally-stabilized VO_2_ thin films in this study presents a promising route to enhance the lifetime, endurance, and reliability of VO_2_-based smart thin-film heterostructure devices.

## Results and Discussion

**Figure 1:**
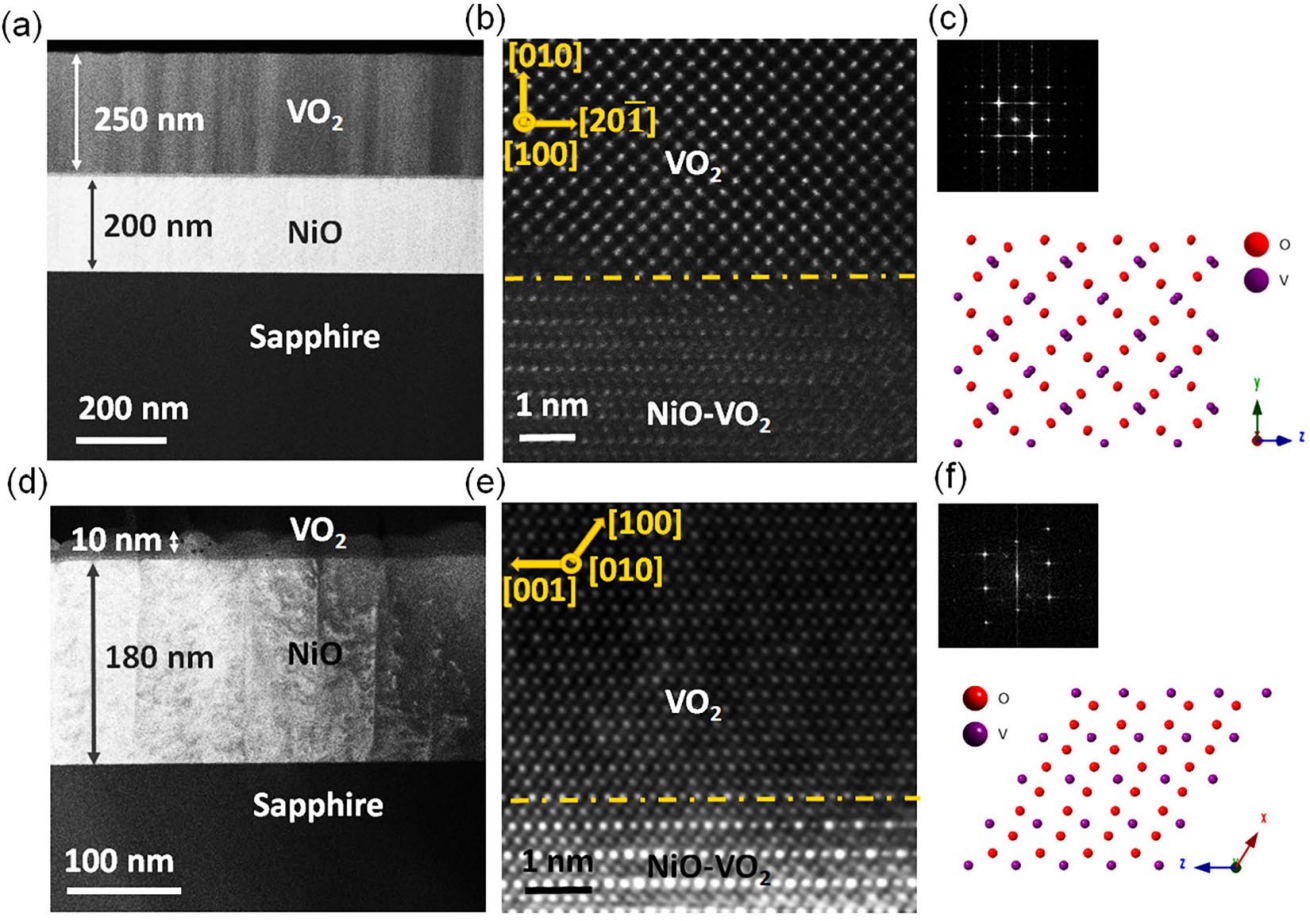
(**a**) The low magnification cross-sectional HAADF image of VO_2_/NiO/(0001)sapphire heterostructures of 250 nm (thick) VO_2_, (**b**) the atomic resolution HAADF image of VO_2_/NiO interface of thick VO_2_ films, and (**c**) the Fourier transform of the VO_2_ thick films and the corresponding atomic model belong to the zone axis of [100] monoclinic phase. (**d**) The low magnification HAADF micrograph of thin VO_2_ films, (**e**) the atomic resolution HAADF image of VO_2_/NiO interface of thin VO_2_ films, and (**f**) the Fourier transform of VO_2_ thin films and the corresponding atomic model belong to the zone axis of [010] monoclinic phase.

To trap the strain uniformly in the VO_2_ thin films, we synthesized epitaxial films below the critical thickness where strain energy is insufficient to trigger the nucleation of misfit dislocations. The dislocation formation occurs as the thickness reaches ~15 nm^10^. The critical thickness calculation is provided in Supplementary. The NiO is used as the buffer layer because the VO_2_ film on top of it can be almost fully relaxed above the critical thickness through domain matching epitaxy (DME) paradigm with near bulk behavior^26–28^. This sample is used as a control sample. The VO_2_/NiO/c-sapphire heterostructures were deposited by pulsed laser deposition (PLD) technique using a 200 nm NiO layer as a buffer. The high angle annular dark field (HAADF) images in Fig. 1 show the thick and thin VO_2_ film heterostructures with VO_2_ film thicknesses of 250 ± 1 nm (Fig. 1a) and 10 ± 1 nm (Fig. 1d), respectively. The atomic resolution images of the films also presented in Fig. 1b and e. There is a 10 nm layer of mixed NiO-VO_2_ at the interface. The Fourier transform of VO_2_ layers and the atomic models of the zone axis belong to HAADF images that provided in Fig. 1c and f for thick and thin film, respectively. Both films were confirmed to be monoclinic from this Figure, and more complementary information presented in Supplementary (Figures S1 and S2). However, two different crystallographic alignments in thin and thick VO_2_ samples were recognized. In the thick VO_2_ film, the planes alignments are NiO(110)$\parallel$ monoclinicVO_2_(001)$\parallel$tetragonalVO_2_(001) and NiO($\bar{2}11\,$)$\parallel$ monoclinic VO_2_(20 $\bar{1}$)$\parallel$ tetragonal VO_2_(100) as in-planes and NiO(111)$\parallel$ monoclinic VO_2_(010) as out-of-planes. While in thin VO_2_ films we have NiO($\bar{2}\,11$)$\parallel$monoclinic-VO_2_(10 $\bar{2}$) and NiO(110)$\parallel$monoclinicVO_2_(010) as in-planes and NiO(111)$\parallel$ monoclinic VO_2_(100) as out-of-plane alignments. The details of plane alignments and DME are presented in Supplementary Table S1^9^. The HAADF image in Supplementary Figure S3a shows the full relaxation of misfit strains within the thick films by dislocation formation every 10/11 planes. In the case of thin samples, no dislocation formation was observed even at the high-magnification HAADF image (Supplementary Figure S3b). This shows that the strain is trapped in the thin film without any relaxation by dislocation.

**Figure 2:**
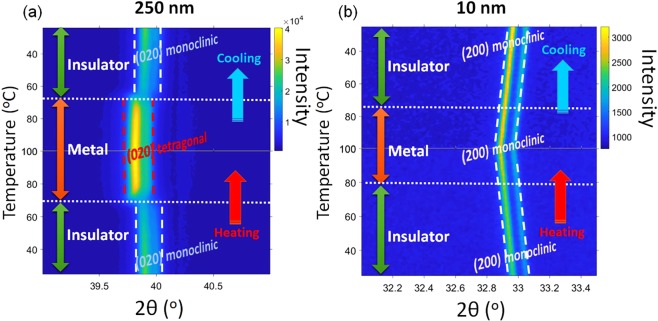
*In-situ* XRD measurements as a function of temperature during heating and cooling cycles of (**a**) thick, and (**b**) thin VO_2_ samples.

The monoclinic nature of both thin and thick films was verified using XRD patterns and Raman spectroscopy, as shown in Supplementary Figures S4 and S5, respectively. The films were heated up and cooled down to monitor the structural changes across the transition, using the *in-situ* XRD diffraction and HAADF imaging^29^. Temperature-dependent XRD measurements presented in Fig. 2a show that the VO_2_ thick films undergo a sharp structural transition from (020) tetragonal phase to (020) monoclinic phase during the transition. The 2θ position and the intensity of the (020) out-of-plane are different in tetragonal and monoclinic crystal structures due to the different d-spacing and structure factors. This leads to a clear shift in the *in-situ* XRD patterns across the monoclinic to tetragonal transition in the thick film of VO_2_. On the other hand, there is no sharp structural transformation observed in thin VO_2_ films with only out-of-plane peak (200) in Fig. 2b. However, thermal expansion/contraction was observed during the heating/cooling cycles. From the XRD out-of-plane d-spacing measurements, the thick film, at room temperature, contains only 0.013 ± 0.001% out-of-plane strain which is attributed to thermal strain during the cooling down cycle. However, from the 2θ position of (200) planes of the thin VO_2_ sample at room temperature, there is 8.624 ± 0.001% out-of-plane strain compared to bulk VO_2_ structure resulting from misfit and thermal strains.

**Figure 3:**
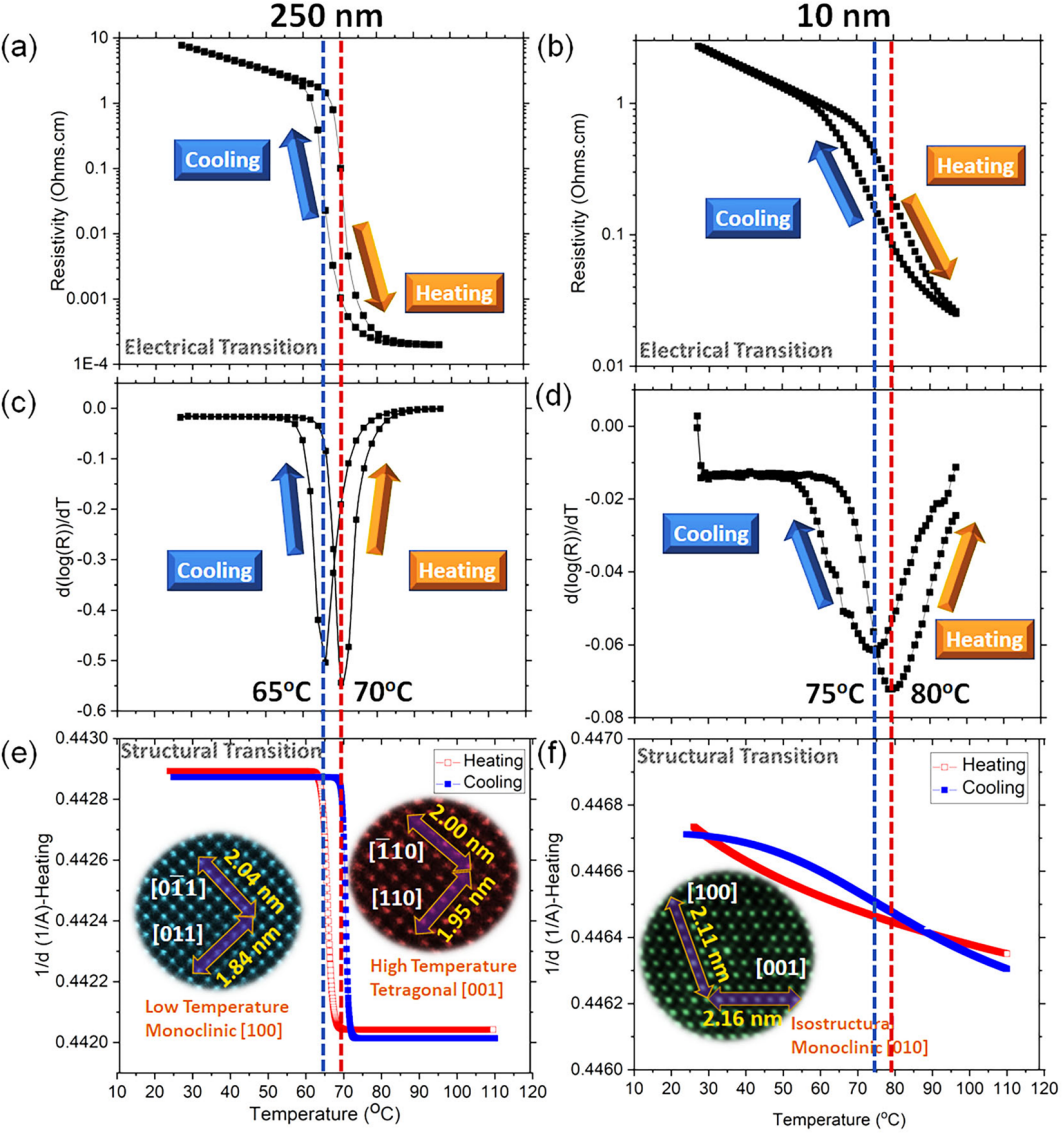
The electrical resistivity as a function of temperature for (**a**) thick, and (**b**) thin VO_2_ samples. The derivative of electrical resistivity versus temperature of (**c**) thick, and (**d**) thin VO_2_ samples. The out-of-plane d-spacing (obtained from *in-situ* XRD measurements) measured as a function of the temperature of (**e**) thin, and (**f**) thick VO_2_ samples. In the inset of the image (**e**), the low and high-temperature HAADF images are provided in the case of the thick sample, and in the inset of the image (**f**), the isostructural HAADF image of the thin sample is presented.

Figure 3 illustrates electrical and structural changes as a function of temperature for thin and thick samples. The structural change has been defined as an abrupt shift in the d-spacing which is extracted from *in-situ* XRD patterns. As is seen from temperature-dependent electrical transport measurements in Fig. 3a,b, both samples undergo a metal-to-insulator transition. The exact value of the metal-to-insulator transition temperatures (T_MIT_) is derived from Fig. 3c,d, the derivative of electrical resistivity versus temperature, for thick and thin samples, respectively. The T_MIT_ for the thick sample is almost identical to what has been reported for bulk VO_2_ (≈68 °C), however, it is increased by 10 degrees (≈78 °C), for the thin sample due to residual in-plane tensile strain along the c-axis^14^. From 1/d vs temperature plots in Fig. 3e,f, in the thick sample, the structural transition is very sharp with an abrupt drop at the transition temperature. However, in the case of the thin sample, there is no abrupt change in 1/d and the gradual thermal expansion and contraction during heating and cooling cycles are indicating no structural transition. In the inset of Fig. 3e, HAADF images at low and high temperatures of thick samples are depicted. The atomic distances after image calibration match with [100] zone axis of the monoclinic (low temperature) and [001] zone axis of the tetragonal (high temperature) structures for the thick sample. The HAADF image of the thin sample (the inset of Fig. 3f) did not show any change at high and low temperatures with [010] zone axis of the monoclinic structure.

**Figure 4:**
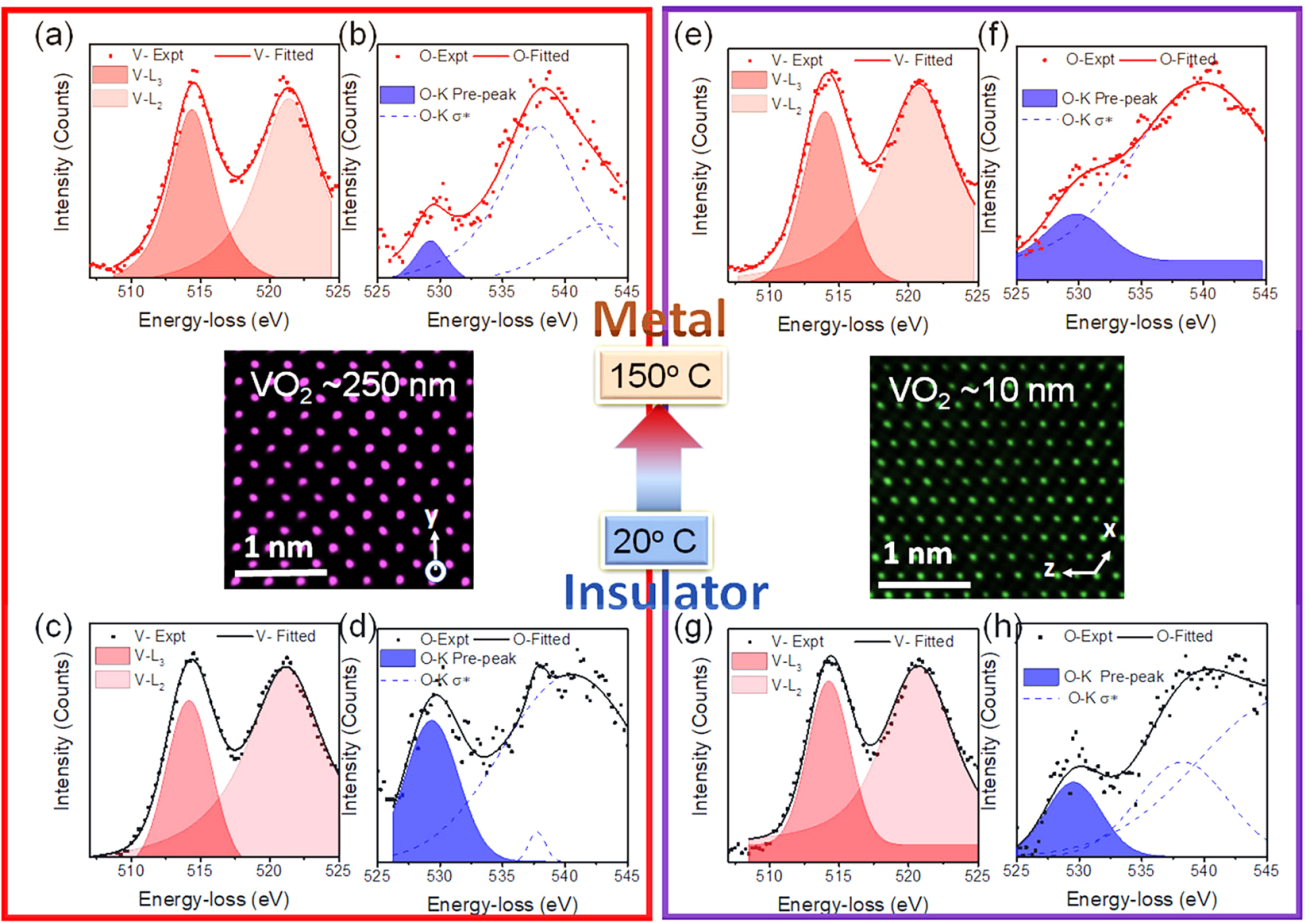
*In-situ* EELS spectra obtained for V-L_2_, _3_ and O-K_1_ edges at high (150 °C) and low (20 °C) temperatures for thick and thin VO_2_ films; the peaks at 513 eV and 521 eV correspond to V-L_3_ and V-L_2_ edges; the peak at 529 eV is indicative of O 1 s electron excitation to dt_2g_ orbitals including π* and ${{\rm{d}}}_{\parallel }$ orbitals. Spectra (**a**,**b**) at 150 °C and (**c**,**d**) at 20 °C represent the thick VO_2_ films. Spectra (**e**,**f**) at 150 °C and (**g**,**h**) at 20 °C represent thin VO_2_ films. The atomic resolution HAADF images corresponding to low-temperature structures of thick and thin films with [100] and [010] zone axis, respectively, are shown in the center of the figure.

We carried out *in-situ* EELS spectroscopy measurements at V-L_32_ and O-K edges in both thin and thick VO_2_ films at ambient (20 °C) and elevated (150 °C) temperatures. We first discuss the shifts/changes in absorption edges (particularly V (L_3_, L_2_) and O-K pre-peak) in the metallic and insulator states of both samples and subsequently compare the results between thin and thick film samples. The intensity axes are all normalized for a comparison. The EEL spectra in Fig. 4a,c,e,g show the peaks at ~513 eV and ~521 eV, corresponding to V-L_3_ and V-L_2_ edges, respectively, which are induced by the excitation of 2p_3/2_ and 2p_1/2_ core electrons to unoccupied d-orbitals near the Fermi level (from 2p^6^3d^1^ to final states of form 2p^5^3d^2^). While, Fig. 4b,d,f,h exhibit O-K edge, constituting of a pre-peak and the main peak (~532 eV), which arises from the excitation of O 1 s (O-K_1_) electrons into 3d bands. Various spectra of V-L_32_ and O-K edges obtained from *in-situ* EELS under different experimental conditions were fitted with Gaussian peaks and are presented in Supplementary Table S2. In the case of thick (~250 nm) VO_2_ film, which represents bulk-like behavior, the intensities of V-L_3_ and V-L_2_ peaks appear unchanged during the transition from insulating (at low-temperature) to metallic (high-temperature) phase, as shown in Fig. 4a,c. The changes in these peaks are possibly unresolved due to the insensitivity of core-electron excitations (2p to 3d) in reflecting the narrow bandgap (0.63 eV) closing with temperature. On the contrary, electron excitations from O 1 s (O-K) into the p band appear to be more sensitive to the changes in the electronic structure during the insulating-to-metal transition. The collected spectra consist of O-K pre-peak (~529 eV) and σ* (~532 eV) peak, where the pre-peak is indicative of O 1 s electron excitation to t_2g_ d-orbitals and includes the information of ${{\rm{d}}}_{\parallel }$*orbital and the π* energy bands. In the experiments, the broadening and the intensity of the pre-peak are carefully monitored, as any variation in pre-peak is reflective of the change in the orbital occupancy, attributing to the insulator to metal transition^1^. See the discussion in Supplementary for the orbital occupancy changes across the transition. While comparing O-pre-peak in Fig. 4b,d, a clear increase in the intensity and FWHM is observed with decreasing temperature (transiting from metallic to insulator phase at room-temperature). This broadening in O-pre-peak of insulating phase happens due to the upshifting of the π* orbital and creation of a bandgap (~0.63 eV) due to d-orbital splitting at 20 °C (below the T_MIT_)^30^. The upshifting and destabilization of the π* band level are due to the V displacement perpendicular to the c-axis (antiferroelectric distortion and formation of the V-V pairs) leading to the perturbation in bonding orbitals. The origin of d-orbital splitting is explained to be either due to homopolar bonding of V-V and c/a ratio changes (Peierls transition)^16^ or through electron-electron interactions (Mott transition)^17^. At 150 °C, ${{\rm{d}}}_{\parallel }$ and π* orbitals are highly overlapped and assist in conducting electrons through the overlapped density of states.

The behavior of V-L_32_ and O-K edges in thin (~10 nm) VO_2_ films across the transition is observed to be largely similar to the thick (~250 nm) films. In the case of the thin film (~10 nm) sample, the intensities of V-L_3_ and V-L_2_ peaks remain unchanged with increasing temperature from 20 °C (Fig. 4g) to 150 °C (Fig. 4e). Furthermore, O-K pre-peak broadens with decreasing temperature, similar to the thick sample. However, the biggest difference is observed in the characteristic absorption edges while comparing the thick and thin samples together. First, the differences between V-L_3_ and V-L_2_ peaks in thick and thin film samples at 6.4 eV and 6.1 eV, respectively, are suggestive of the strained structure of isostructural monoclinic phase in thin VO_2_ film^9^. Second, one finds the O-K pre-peak to be broader in the thin sample at room temperature. This suggests the overlapping of energy bands in the atomic structure and a shrunken bandgap through a further upshifting of π* due to structural modification in the thin films. While comparing O-pre-peak of thick and thin VO_2_ films at a higher temperature (150 °C), O-K pre-peak in the thin case again appears broader due to upshifting of π*. This leads to a lower conductivity in the thin sample at the metallic stage as compared to the bulk since the conductivity is coming only from the narrow ${{\rm{d}}}_{\parallel }$ orbital electrons. This is consistent with the resistivity measurements presented in Fig. 3.

To understand the origin of insulator-to-metal transition difference in thermally stable isostructural monoclinic VO_2_ than thick VO_2_, DFT calculations were performed. To account for strong electron correlations, we employed the simplified Hubbard *U* = 3.10 eV scheme on the V 3*d*-states^31^. This value estimates a bandgap of 0.63 eV for the nonmagnetic monoclinic phase (*NM-M1*), which is consistent with prior experimental values of 0.6–0.7 eV^32–34^ and other electronic structure calculations^24,34–37^. In all cases, we examined both nonmagnetic (*NM*) and ferromagnetic (*FM*) monoclinic structures with space group *P*2_1_*/c*. For FM calculations, the magnetization was initiated with (+1) on each V ion with a total magnetization of 5 to allow for self-consistent convergence. Table 1 lists the structural parameters for the fully optimized *NM* and *FM* structures obtained with this approach as well as those from previous hybrid calculations^24^ and corresponding experiments^38,39^. Similar to Xu *et al*.^24^, we find that our fully optimized *NM* monoclinic structure (*NM-M1*) is in good agreement with the experimental monoclinic structure, while our FM monoclinic structure (*FM-M0*) is in good agreement with a recent report of a metallic monoclinic (*mM*) phase. Unlike previous hybrid functional calculations^24^, we find that the *FM-M0* phase is higher in energy than the NM-M1 phase by 0.013 eV/f.u. (We note that larger values of *U* function to both increase the NM-M1 bandgap and increase the energetic differences between the *FM-M0* and *NM-M1* phases while having minimal effects on the structural parameters. For example *U* = 4 eV increases the *NM-M1* bandgap to 0.85 eV and favors the *FM-M0* phase by 0.098 meV/f.u.).

**Table 1:** Lattice constants, V-V bond angles and lengths, and dimerization difference Δ_*V*−*V*_ for VO_2_ groundstate nonmagnetic (NM-M1) and ferromagnetic (FM-M0) phases. Comparisons to previous experiment and theory are included for validation purposes.

		*NM-M1*	*M1*	*NM-M1*	*FM-M0*	*mM*	*FM-M0*
		This work	Expt.^43^	Theory^24^	This work	Expt.^44^	Theory^43^
*a* (Å)		5.53	5.75	5.53	5.69	5.69	5.59
*b* (Å)		4.57	4.54	4.51	4.55	4.59	4.50
*c* (Å)		5.38	5.38	5.28	5.34	5.29	5.29
*β* (°)		121.54	122.65	121.93	121.95	122.61	122.05
V-V angle (°)		165	168	166	172		175
V-V bond (Å)	short	2.45	2.62	2.44	2.71	2.72	2.69
	long	3.13	3.17	3.14	2.99	2.98	2.94
Δ_*V*−*V*_ (Å)		0.68	0.55	0.70	0.28	0.26	0.25

To better understand the metal-insulator transition we examined the effects of strain on the NM bandgap. Here, we fixed the in-plane lattice constants for the NM phase to those obtained from experiment assuming a V-V angle of 122° for both the low temperature (NM-M1_low_; *a* = 5.797 Å, *c* = 5.427 Å) and high-temperature phases (NM-M1_high_ *a* = 5.797 Å, *c* = 5.460 Å). (See Supplementary Table S3 for further structural details). In both cases, we fully relaxed the out -of -plane lattice constants; obtaining 4.452 Å and 4.437 Å) for NM-M1_low_ and NM-M1_high_, respectively. However, both structures gave a bandgap of 0.69 eV – not a significant deviation from the optimized NM-M1 value of 0.63 eV.

**Figure 5:**
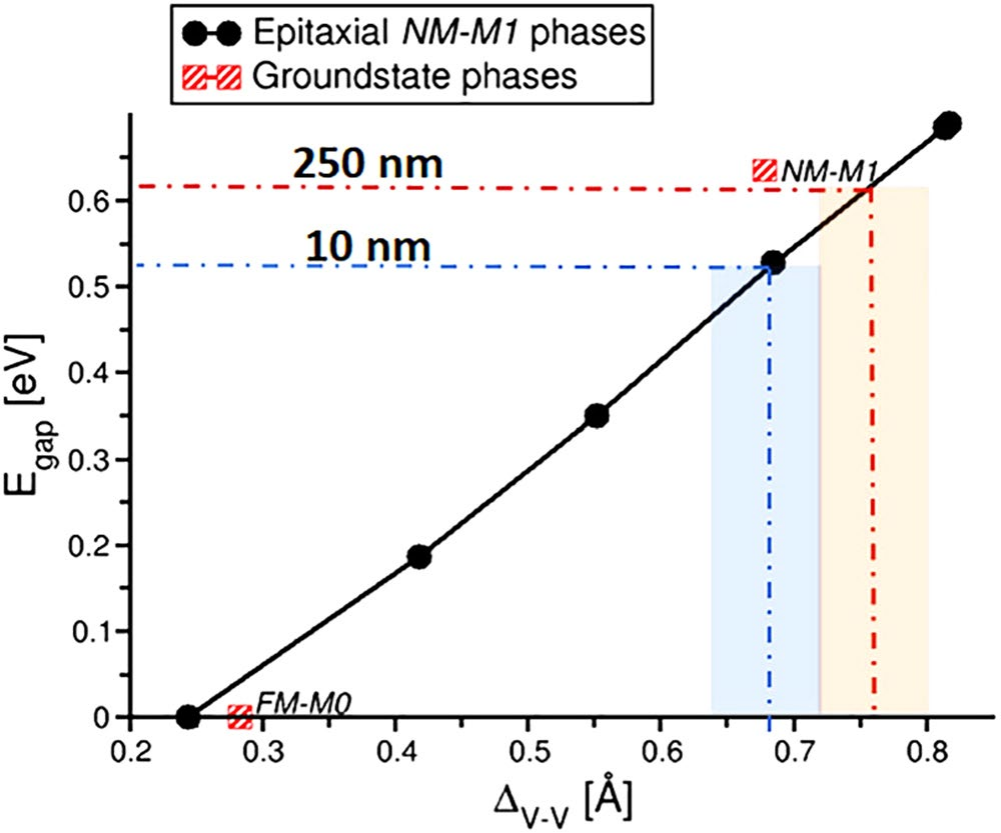
Bandgap (E_gap_) as a function of the difference between short and long V-V bonds (Δ_*V*−*V*_) in VO_2_. Black solid circles indicate data obtained from structures fixed to the epitaxially constrained experimental thin film values. Red hashed squares indicate the groundstate phases: NM-M1 (nonmagnetic M1 monoclinic phase) and FM-M0 (ferromagnetic M0 monoclinic phase). FM phase’s bandgaps were obtained from NM calculations of the FM groundstate atomic configurations.

Noting the large difference in V-V short and long bonds (Δ_*V*−*V*_) in the FM and NM structures, we investigated the role of V-V dimerization in tuning *E*_gap_. Figure 5 depicts the relationship of *E*_gap_ versus Δ_*V*−*V*_. For this study, we used structures obtained from NM-M1_high/low_ calculations, NM calculations using the atomic coordinates of fully optimized FM structures (with in-plane lattice constants fixed to the low and high temperature phase values) and interpolated intermediate (fixed) structures with Δ_*V*−*V*_= 0.42 Å, 0.55 Å and 0.69 Å (Table 2). Here, we see *E*_gap_ systematically decreases with decreasing Δ_*V*−*V*_; and ultimately leading to a metallic transition at Δ_*V*−*V*_ = 0.24 Å. Interestingly, this value is close to that of Δ_*V*−*V*_ in the fully optimized FM structure. Similar behavior was observed for the MoCl_3_ layered compound where a loss in the Mo-Mo dimerization at high temperature was correlated with an insulator-metal transition^40^. It is also reported that direct bandgap in Ge can be reduced through strain engineering when tetragonal Ge thin films grow below critical thickness on a Si substrate^41^. The fact that the M1 insulator-metal transition may be correlated with a reduction in Δ_*V*−*V*_ similar to the ferromagnetic phase would be consistent with the disappearance of dimerization in the VO_2_ rhombohedral phase.

**Table 2:** Lattice constants, V-V bond angles, and lengths, dimerization difference (Δ_*V*−*V*_), and bandgap (E_gap_) for VO_2_ structures used to explore the effects of dimerization on bandgap.

		*NM-M1*	*NM-M1*	*NM-M1*	*NM-M1*	*NM-M1*	*FM-M1*
		low	high	Δ_*V*−*V*_ = 0.69 Å	Δ_*V*−*V*_ = 0.55 Å	Δ_*V*−*V*_ = 0.42 Å	low
*a* (Å)		5.797	5.797	5.797	5.797	5.797	5.797
*b* (Å)		4.452	4.437	4.429	4.424	4.419	4.483
*c* (Å)		5.427	5.460	5.460	5.460	5.460	5.427
V-V angle (°)		166	166	168	170	172	175
V-V bond (Å)	short	2.58	2.58	2.64	2.70	2.76	2.84
	long	3.39	3.40	3.32	3.25	3.18	3.09
Δ_*V*−*V*_ (Å)		0.81	0.82	0.68	0.55	0.42	0.24
E_gap_ (eV)		0.69	0.69	0.53	0.35	0.19	0.00

Figure 5 shows the approximate bandgap and Δ_*V*−*V*_ corresponding to thin and thick VO_2_ films, based on resistivity vs temperature measurements. From the resistivity plots, one can deduct the ratio of bandgap values from lnρ vs 1/T plots (where ρ is conductivity, and T is temperature), to be equal 1.243 for E_g thick_/E_g thin_. This ratio provides the corresponding Δ_*V*−*V*_ for thin and thick VO_2_ films as shown in Fig. 5. The decrease in bandgap due to smaller Δ_*V*−*V*_ facilitates the insulator to metal transition without going through the structural transition. Since the calculations show that the reduction in bandgap can be promoted through the introduction of ferromagnetic nature in the monoclinic phase, the temperature-dependent magnetic characterization of the films were obtained (see Supplementary Figure S6), which shows evidence of ferromagnetism and thus long-range ferromagnetic ordering^42^.

It is hypothesized that the insulator-to-metal transition in the isostructural monoclinic VO_2_ films is a combination of both electron-electron and phonon-electron interactions, which is tuned by the bandgap modification. In this case, since we have modified the V-V bond length ((Δ_*V*−*V*_is smaller) in the thin VO_2_ films, the resulting narrowed bandgap facilitates the electron-electron interactions, and leads to the insulator-to-metal transition without any structural change with temperature. The presence of Δ_*V*−*V*_ perturbs the π* orbital and makes it narrower due to shorter V-O bonds perpendicular to c-axis, which tends to lift the π* above the Fermi level. As a result, conductivity is only due to the narrow unoccupied portion of the ${{\rm{d}}}_{\parallel }$ band. Also, the ${{\rm{d}}}_{\parallel }$ orbital is not degenerate due to the monoclinic structure (presence of a small bandgap, between ${{\rm{d}}}_{\parallel }$ and ${{{\rm{d}}}^{\ast }}_{\parallel }$) at a high-temperature. The orbital non-degeneracy can be also inferred from dimerization and broadening of the O-K _π*_ for the thin and thick sample, shown in Fig. 4f and b, respectively. This phenomenon was predicted by Goodenough for the distorted oxygen octahedra system where localized holes have been added to VO_2_^6^.

**Figure 6:**
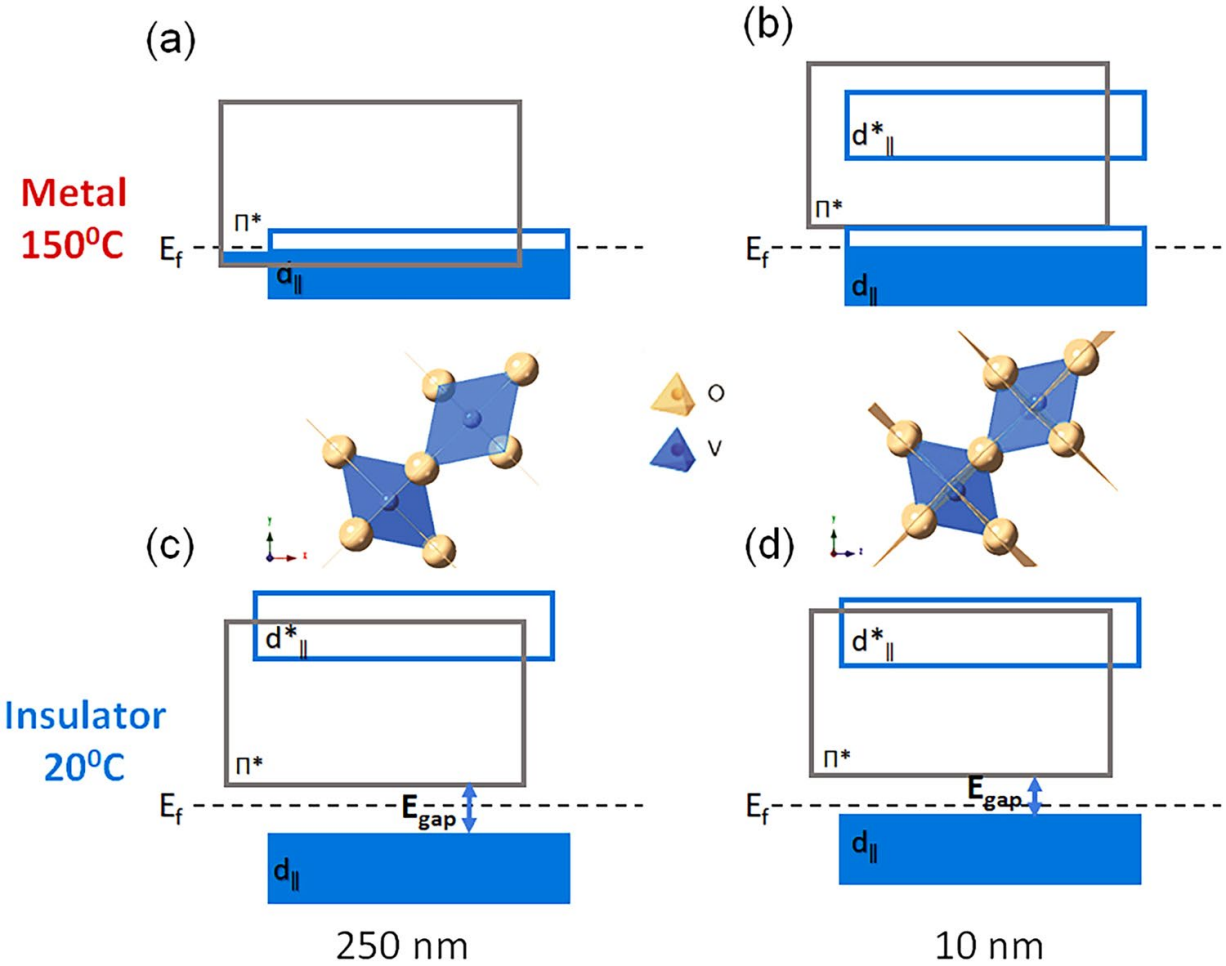
Schematic of VO_2_ band structure in the metallic state for (**a**) thick and (**b**) thin samples, schematic of VO_2_ band structure in the insulating state for (**c**) thick and (**d**) thin samples. The oxygen octahedra are shown in the center of the figure for high-temperature structures of thick and thin films; the vanadium atom displacement is depicted from the center of the octahedron in the case of thin samples due to strain pinning.

Based on the temperature-dependent EELS analyses, resistivity measurements, and DFT calculations, a suggestive band structure diagram of both thin and thick VO_2_ films at temperatures across the MIT are illustrated in Fig. 6. At high temperature, the π* orbital is perturbed and shifted higher energy level in the thin sample (Fig. 6b) compared to the thick sample (Fig. 6a). This upshift is due to the non-cooperative displacement of vanadium ions from the center of the octahedra, which results in V-V dimerization (with the presence of Δ_*V*−*V*_). At low temperature (Fig. 6c,d), the thin sample has less crystal field splitting in both V-L and O-K edges with smaller bandgap than the thick case.

It is shown that electrical and structural transitions in the thick VO_2_ film are strongly-correlated through electron-electron interactions between O 2p and V 3d states, which is not the case for the thin strained films. In unstrained VO_2_ systems, the lowering of crystal symmetry from tetragonal to monoclinic is attributed to the lift of orbital degeneracy, leading to typical bandgap evolution. It is suggested that the electronic structure in the strained monoclinic VO_2_ film (10 nm thick) is strongly modified as compared to the relaxed thick film, resulting in a considerable bandgap narrowing. This is driven by decreasing Δ_*V*−*V*_ in the strained ferromagnetic monoclinic VO_2_ thin films. This modified structure with lowered bandgap allows the transition to follow Mott physics^43^. At low temperatures below the transition, there are fewer electrons occupying the Fermi level, causing the insulating behavior while simultaneously reducing the overall resistivity of the films. This is also observed experimentally in the present study in the temperature-dependent resistivity measurements. With increasing temperature above the transition temperature, electron concentration rises and closes up the bandgap to form a complete metallic state of monoclinic phase. Overall, we illustrate that the positive impact of all the factors contributing to the formation of the structurally-pinned monoclinic VO_2_ film will lead to the technologically improved smart sensing and switching devices. The present process allows wafer-scale integration of VO_2_ device layers on sapphire using NiO buffer layer, and it has been extended to silicon wafers utilizing NiO/YSZ/Si(100) heterostructures^28^.

## Conclusion

We have shown that by decreasing Δ_*V*−*V*_ through misfit strain in epitaxial monoclinic VO_2_ thin-film heterostructures, the bandgap decreases in a way that structural transition is pinned, while electrical transition occurs. To trap the strain, VO_2_ films were grown pseudomorphically below the critical thickness where no strain releasing dislocations are formed for lattice relaxation. It is envisaged that in the strain-trapped thin films at high temperature, the oxygen octahedra are distorted to the point where vanadium ions are not stable in the center of the octahedra and form short and long V-V bonds, which destabilize the bonding d orbitals. This destabilization affects the bonding and nonbonding orbitals stability and their occupancy which accordingly locks the structural changes over the transition and changes the bandgap and the conductivity of the high-temperature phase. This study opens up the opportunity to lock other degrees of freedom including spin, orbital, and charges to control critical aspects of transition in VO_2_ thin films.

## Methods

### Thin Film Growth

A KrF excimer laser (Lambda Physik) with 248 nm wavelength and a pulse duration of 25 ns was used to ablate high-quality VO_2_ and NiO targets^44,45^. The whole system is connected to a vacuum chamber with a base pressure of 0.5 µTorr and is connected to gas inlet allowing adjustment of oxygen partial pressure. The c-sapphire substrates were cleaned and mounted inside the vacuum chamber. The cleaning procedure includes acetone-vapor cleaning for 10 min and then substrates were ultrasonicated in methanol for 15 min followed by deionized water ultrasonication for 20 min. The substrates were then dried by a high purity nitrogen gun before loading into the vacuum chamber. The NiO films were grown at 700 °C for 3500 pulses under 0.1 mTorr of oxygen pressure. The VO_2_ films were grown at 550 °C for 200 and 3500 pulses for thin and thick samples, respectively, under 12 mTorr of oxygen pressure. The energy density and frequency were set at 3–3.5 J/cm^3^ and 5 Hz, respectively. The samples were cooled down under the same oxygen pressure.

### *In Situ* X-ray Diffraction Measurement

The *in-situ* XRD measurements were carried out on a PANalytical Empyrean diffractometer using Cu-K_α_ radiation. This technique was used to determine *in-situ* structural changes during heating and cooling cycles performing in the 25–120 °C range. The diffraction data were acquired using a step size of 0.013° 2θ and the count time of 1 S per step.

### Scanning Transmission Electron Microscopy

The samples were prepared using a combination of mechanical polishing and FIB lift-out methods using an FEI Helios DualBeam Focused Ion Beam operating at 2–30 KeV. The samples were mounted on an electrical chip to be able to increase the temperature during the experiment. The details about sample preparation have been published elsewhere^29^. The FEI Titan 80–300 probe aberration-corrected scanning transmission electron microscope (STEM) operated at 200 KV, was employed to collect HAADF images. The electron energy loss spectra (EELS) were also collected across the interfaces operating at 200 KeV with a 28 mrad collection angle, 19.6 mrad convergence angle, and a 2.5 mm EELS entrance aperture. Spectra were acquired using a 0.25 eV ch^−1^ dispersion with a ∼35 pA probe current and 0.07 nm pixel size to perform elemental analysis of V-L_2,3_ (513, 521 eV), O-K_1_ (529, 532 eV), and Ni-L_2,3_ (855, 872 eV). A peak fitting routine was performed on V-L and O-K edges using Gaussian profiles for detailed analysis^46–48^.

### Electrical Measurement

The electrical transport measurements were collected by physical property measurements system (PPMS) using the Van der Pauw method by Quantum Design over the temperature range of 25–120 °C during heating and cooling cycles. The resistivity data were collected using a step size of 1 °.S^−1^ at zero magnetic fields.

### Magnetic Measurement

The magnetic properties were measured using a vibrating superconducting quantum interference device (SQUID) magnetometer^49–51^.

### Density functional theory calculations

Density functional theory calculations were performed using the Quantum Espresso simulation package (v6.2)^52^. In all cases, a Monkhorst-Pack gamma centered 6 × 6 × 6 *k*-point grid was employed with energy cutoffs of 80 and 800 Ry for the plane waves and charge density, respectively. Exchange and correlations were approximated using the modified Perdew-Burke-Erzenhoff functional designed specifically for solids (PBEsol)^53^. V (3 *s*^2^ 3*p*^6^ 4 *s*^2^ 3*d*^3^) and O (2 *s*^2^ 2*p*^4^) pseudopotentials from the Garrity-Bennet-Rabe-Vanderbilt (GBRV) high-throughput pseudopotential repository were used^54^.

## Supplementary information

Supplementary Information
