# Supplementary material for: Electrical Transition in Isostructural VO2 Thin-Film Heterostructures
Source: Sci Rep. 2019 Feb 28;9:3009. doi: 10.1038/s41598-019-39529-z (PMC6395818; doi:10.1038/s41598-019-39529-z)
Supplement: Supplementary file 1 — Supplementary Information [file 41598_2019_39529_MOESM1_ESM.pdf]

Supplementary information

Electrical Transition in Isostructural VO_2 Thin-Film Heterostructures

Adele Moatti[a], Ritesh Sachan*[a, b], Valentino Cooper[c], Jagdish Narayan[a]*

[*] Equal contribution as the first author

[a] Materials Science and Engineering, North Carolina State University, Raleigh, North Carolina 27606
[b] Materials Science Division, Army Research Office, Research Triangle Park, North Carolina 27709
[c] Materials Science and Technology Division, Oak Ridge National Laboratory, Oak Ridge, TN 37830

Email: amoatti@ncsu.edu, rsachan@ncsu.edu

Keywords: VO_2 thin films, orbital occupancy, Mott transition, Peierls transition, Isostructural transition

The critical thickness calculation

The critical thickness (h_c) at which it becomes energetically favorable for a thin film to contain dislocations:

$$h_c = \frac{b(1 - \upsilon cos^2\theta)sin\theta cos\emptyset}{4\pi(1+\upsilon)\varepsilon_0}\ln(\alpha h_c/b)$$

Where b is the magnitude of Burgers vector of the dislocation, υ is the Poisson's ratio, θ is the angle between Burgers vector and dislocation line, ϕ is the angle between the normal of the dislocation plane and film plane, and α is the dislocation core radius factor which varies with strain, ε_0.

The slip systems in the VO_2 monoclinic structure are $1/2[0\bar{1}1](011)$ and $1/2[10\bar{1}](101)$. So Burgers vector calculated to be 1.749 A and 1.553 A, respectively.

$$h_c = \frac{0.048b}{\varepsilon_0} ln(\alpha \frac{h_c}{b})$$

Thus, for 10nm thick film, the critical thickness calculated to be ~15 nm. The role of tensile strain has been explained to increase the kinetic barrier for dislocation nucleation. Even though thermodynamically

the critical thickness is calculated at 15 nm, kinetically the dislocation formation is not happening up to higher values.

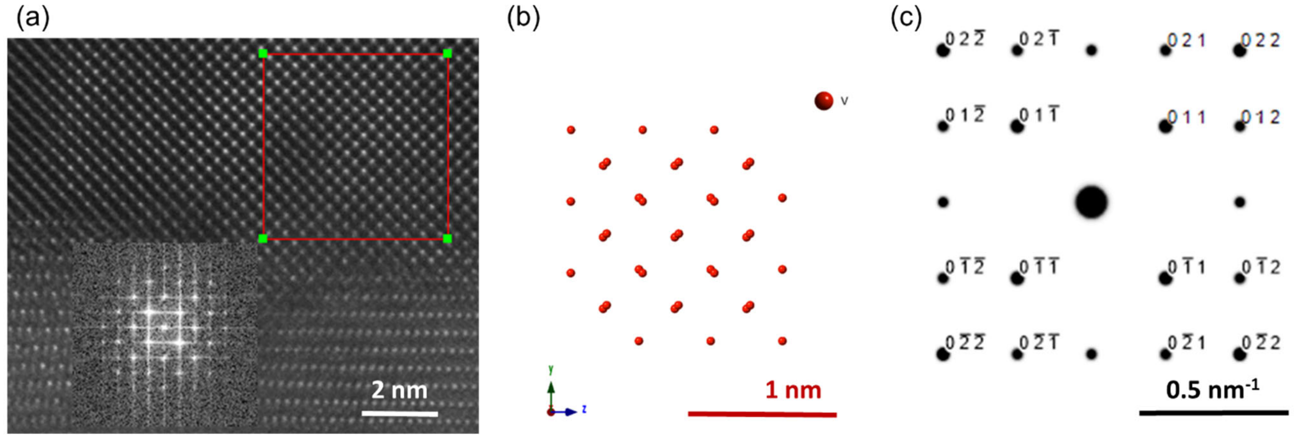

Figure S1. a) The HAADF image and its FFT belong to the thick VO_2 film with the zone axis of [100] monoclinic, b) the corresponding atomic alignment, and c) the indexed simulated pattern for the mentioned zone axis in the monoclinic VO_2 structure

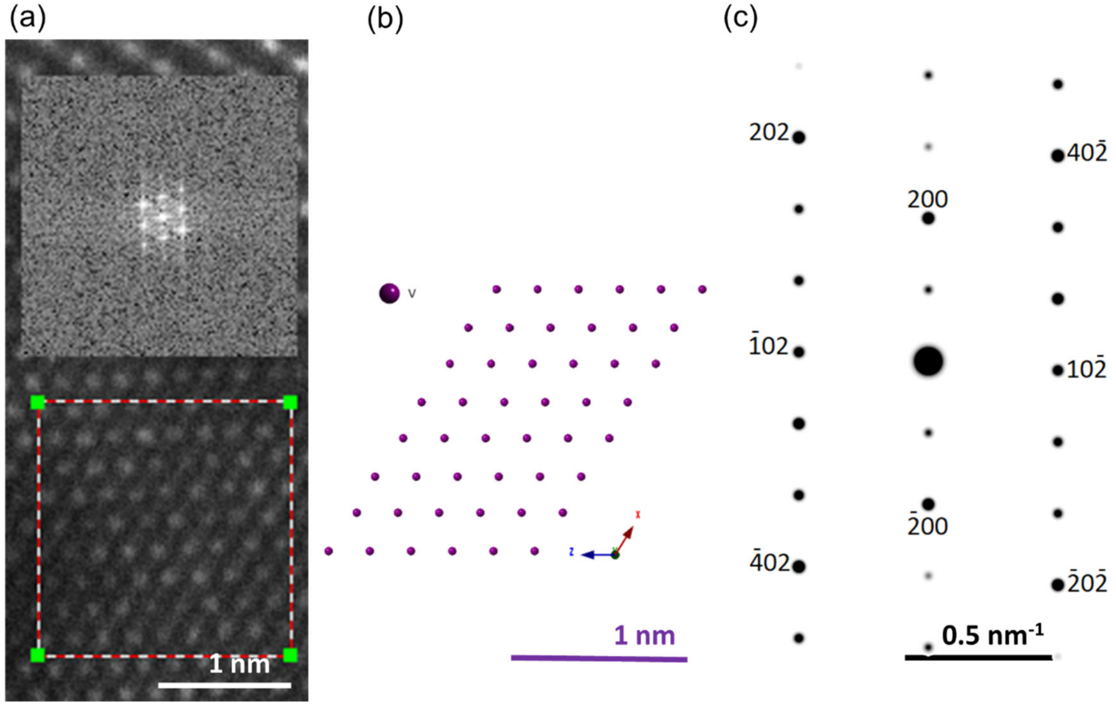

Figure S2. a) The HAADF image and its FFT belong to the thin VO_2 film with the zone axis of [010] monoclinic, b) the corresponding atomic alignment, and c) the indexed simulated pattern for the mentioned zone axis in the monoclinic VO_2 structure

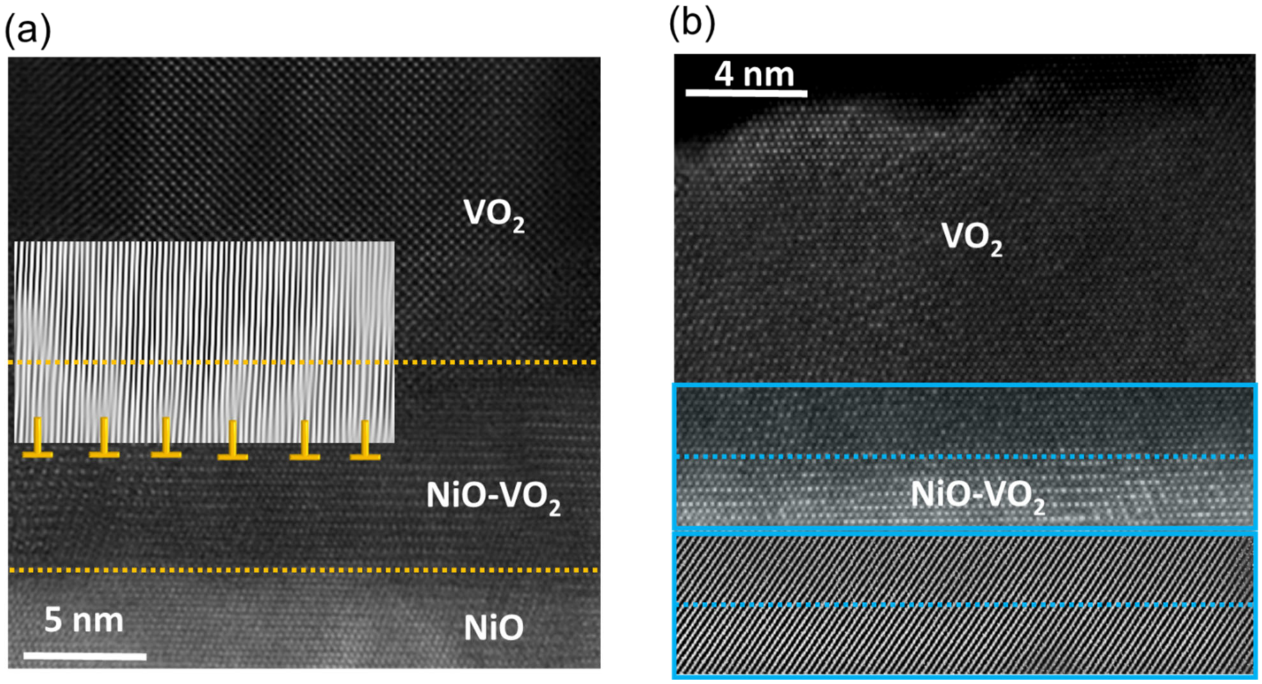

Figure S3. a) The HAADF image and inverse FFT at the interface of the thick NiO/VO_2 film, b) the HAADF image and inverse FFT at the interface of the thin NiO/VO_2 film

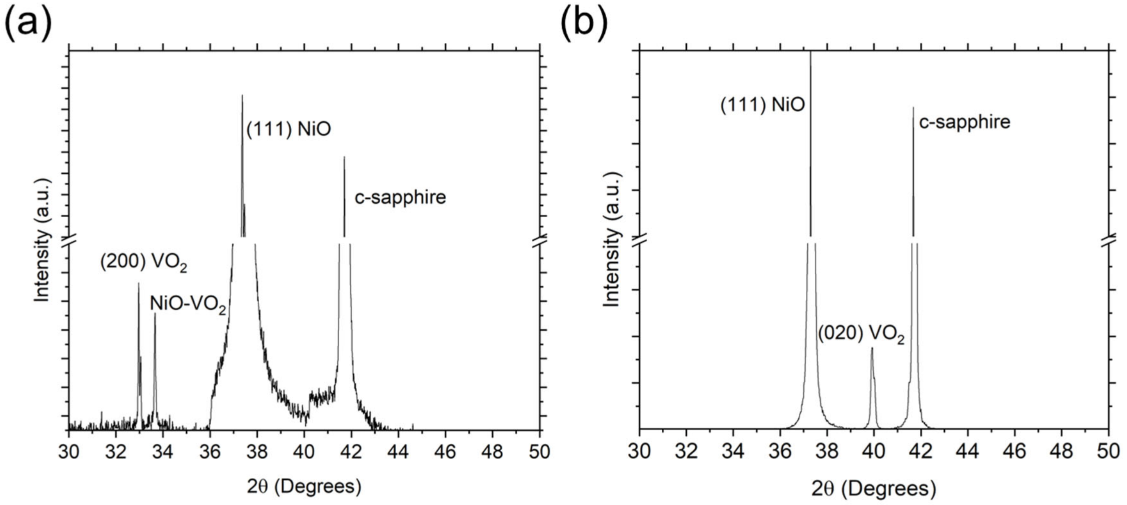

Figure S4. X-Ray diffraction of (a) thin VO_2, and (b) thick VO_2 films.

Raman characterization

To further verify the room temperature VO_2 phase formed in thick and thin films, Raman spectroscopic studies were performed, as provided in **Figure S5**. Both the spectra illustrate the main characteristic peaks of the monoclinic structure of VO_2 at 195 cm^{-1}, and 225 cm^{-1} (assigned to V-V modes), and 618 cm^{-1}, and 625 cm^{-1} (assigned to V-O modes) [1,2], thus confirm the stable monoclinic phase formation in thick and thin VO_2 films. The peak splitting around 620 cm^{-1} in both samples, indicates the oxygen ions are connected to two vanadium ions with a different bond-length along the c-axis. In an interesting observation, however, the V-O peaks are shifted toward lower frequencies in the case of thin VO_2 samples. These shifts are indicative of V-O shorter bond length due to oxygen octahedral distortion. Also, the intensity of V-V modes has changed in thin VO_2 films compared to thick ones which can be inferred that the pairing and tilting of vanadium ions have changed attributed to the change in V-V bond length while maintaining the monoclinic structure. The presence of metallic monoclinic phase stabilized under high pressure has been shown by Raman spectroscopy previously, the result of which is consistent with the present study [3].

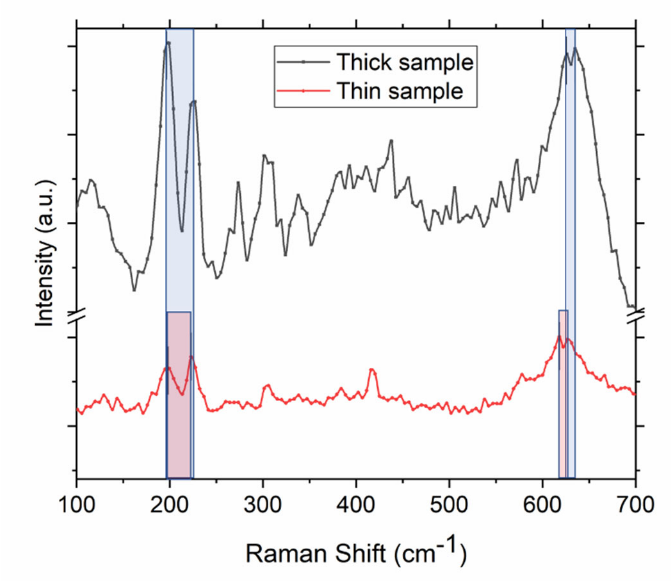

Figure S5. Room temperature Raman spectroscopy of thin and thick VO_2 samples with the characteristic peaks belong to the monoclinic phase as indicated.

Orbital occupancy across the metal-to-insulator transition

During the metal-to-insulator transition, the V^{4+} cation moves away from the center of the oxygen octahedron toward the edges and form V-V pairs and the conductivity decreases sharply. At the high-temperature tetragonal state, V^{4+} in the octahedra obstructs the free rotation of the electrons and quenches the orbital angular momentum giving rise to the splitting of the $3d^1$ energies into e_g ($d_{3x^2-r^2}$, d_{yz}) and t_{2g} ($d_{z^2-y^2}$, d_{xy}, d_{zx}) orbitals [4]. The e_g wave functions are pointing toward O^{2-} thus having higher energies, while t_{2g} pointing between them [5]. Covalent mixing between the two electrons in d_{xy} and d_{zx} and p_π orbitals of the anion results in the formation of the narrow antibonding π* and a wider bonding π band. The remaining electron is nonbonding and goes into $d_{z^2-y^2}$ which is directed parallel to the c-axis. This electron is not involved in the V-O bonding and provides V-V bonding along the c-axis, also partially fills the $d_{z^2-y^2}$ which is the reason for rutile phase being metallic and reduces the c/a ratio. The relative energies and stabilities of the $d_{z^2-y^2}$ and

d_{xy}, d_{zx} orbitals depend upon the c/a ratio as proposed by Goodenough [6], discussed by Hearn [7], and experimentally showed by Aetukuri [8]. We believe that the oxygen octahedra in uniformly strained thin films at high temperature are distorted which means vanadium is not stable in the center of the octahedra and thereby stabilizes the antiferroelectric distortion (the first necessary component of the transition) introduced by Goodenough [6]. According to this theory, the requirement for the insulating band structure is i) destabilizing the π* orbital and/or stabilizing the bottom half of the $d_{z^2-y^2}$ orbital by raising the π* orbital energy above the Fermi level, and ii) splitting of the $d_{z^2-y^2}$ orbital. Goodenough proposed that the distortion of the structure by displacement of V ions perpendicular to c-axis destabilizes the π* orbital (the antiferroelectric distortion) and a decrease in the c/a ratio stabilizes the bottom half of the $d_{z^2-y^2}$ orbital which fulfills the first requirement, and a homopolar V-V bonding along the c-axis split the $d_{z^2-y^2}$ and defines the energy gap [6]. However, Zylbersztejn and Mott believed the role of distortion is to destabilize the π* orbital and to induce the transition and not to determine the electrical gap [9]. They proposed that each V^{4+} has a moment and the energy to form a carrier (defined as band gap) is equal to $U-1/2(B_1+B_2)-J_H$, where U is the Hubbard intra-atomic correlation energy, B_1 and B_2 are the bandwidth of upper and lower Hubbard bands here for the motion of electron (V^{3+}) and hole (V^{5+}), and J_H is the coupling energy. Thus, the bandgap is mainly a correlation gap.

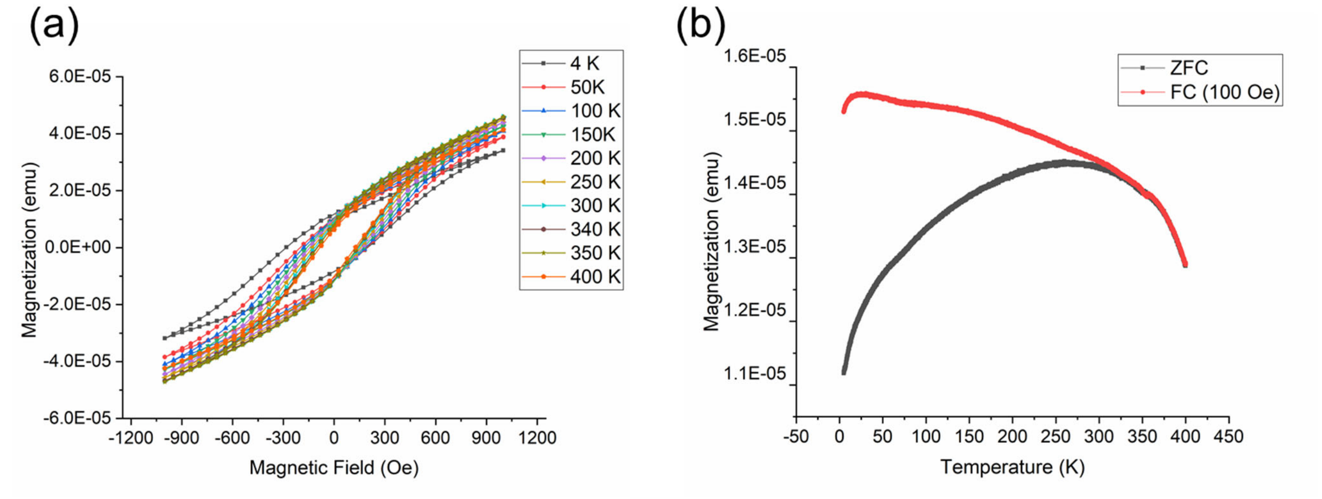

Figure S6. (a) The magnetization vs field plots at different temperatures for thin VO_2 samples and (b) temperature dependence of 100 Oe cooled field (CF) and zero cooled fields (ZFC) magnetization plots of the thin sample

Table S1. The plane alignments, DME paradigm [10], and the strains reported for thin and thick VO_2 films.

	In plane alignments	[1]Misfit Strain %	[2]DME calculations:	Out of plane alignment	Residual out-of-plane Strain %	[3]Residual in-plane Strain %
10 nm VO_2 film	NiO($\bar{2}$11)‖M-VO_2(10$\bar{2}$)	-4.2	23/24, α=0.53	NiO(111)‖VO_2(200)	8.624±0.001	-7.392
	NiO(110)‖M-VO_2(010)	-2.1	47/48, α=0.82			
250 nm VO_2 films	NiO(110)‖M-VO_2(001)‖T-VO_2(001)	3.3	29/30, α=0.67	NiO(111)‖VO_2(010)	0.013±0.001	-0.011
	NiO($\bar{2}$11)‖M-VO_2(20$\bar{1}$)‖T- VO_2(100)	9.9	10/11, α=0.93			

[1]misfit strains are calculated based on tetragonal VO_2 phase for 250 nm films. T and M stands for tetragonal and monoclinic, respectively (When the films transform from tetragonal to monoclinic during the cooling, the dislocations are already formed following the tetragonal or high temperature phase misfit strains.)

[2]Number of alternating planes matching across the interface, and α is the frequency factor.

[3]Poisson ratio of VO_2 thin films is considered to be 0.3.(ref: Thermodynamics of strained vanadium dioxide single crystals)

Table S2. The peak positions and width of the EELS edges for thin and thick VO_2 films at room temperature (RT) and high temperature (HT). The standard deviation in all the measurements was 0.1 eV.

	VO_2 (250 nm)				VO_2 (10 nm)			
	RT		HT		RT		HT	
	Peak position (eV)	Width (eV)	Peak position (eV)	Width (eV)	Peak position (eV)	Width (eV)	Peak position (eV)	Width (eV)
V-L3	514.4	3.2	514.5	3.4	514.3	3.3	514.3	3.6
V-L2	520.8	5.1	520.8	5.2	520.4	5.1	520.8	5.3
O-K (π*)	529.4	4.7	529.3	3.7	529.6	4.6	529.8	4.4

Table S3. Atomic Structures (lattice vectors in Å; atomic positions in relative coordinates):

NM-M1:

a	5.526795	0	-0.00013
b	0	4.566773	0
c	-2.81085	0	4.579404
V	0.234492	0.975573	0.030351
V	0.765508	0.024427	0.969649
V	0.765509	0.475573	0.469649
V	0.234491	0.524427	0.530351
O	0.107259	0.2174	0.213241
O	0.892741	0.7826	0.786759
O	0.892741	0.7174	0.286759
O	0.107259	0.2826	0.713241
O	0.401269	0.699094	0.305006
O	0.598731	0.300906	0.694994
O	0.598731	0.199094	0.194994
O	0.401269	0.800906	0.805006

FM-M0:

A	5.691033	0	0.041931
B	0	4.551353	0
c	-2.86001	0	4.512081
V	0.246348	0.996014	0.009994
V	0.753652	0.003986	0.990006
V	0.753652	0.496014	0.490006
V	0.246348	0.503986	0.509994
O	0.102386	0.205785	0.207984
O	0.897614	0.794215	0.792016
O	0.897614	0.705785	0.292016
O	0.102386	0.294215	0.707984
O	0.399526	0.69787	0.302744
O	0.600474	0.30213	0.697256
O	0.600474	0.197871	0.197256
O	0.399526	0.802129	0.802744

NM-M1 (low):

a	5.79684	0	0
b	0	4.451768	0
c	-2.87591	0	4.602425
V	0.23048	0.974878	0.030438
V	0.76952	0.025122	0.969562
V	0.76952	0.474878	0.469562
V	0.23048	0.525122	0.530438
O	0.103839	0.223491	0.206731
O	0.896161	0.776509	0.793269
O	0.896161	0.723491	0.293268
O	0.103839	0.276509	0.706731
O	0.404299	0.703999	0.306964
O	0.595701	0.296001	0.693036
O	0.595702	0.203999	0.193036
O	0.404298	0.796001	0.806964

NM-M1 (high):

a	5.79684	0	0
b	0	4.43667	0
c	-2.89331	0	4.630266
V	0.230687	0.975217	0.030932
V	0.769313	0.024783	0.969068
V	0.769313	0.475218	0.469068
V	0.230687	0.524782	0.530932
O	0.103072	0.224784	0.205209
O	0.896928	0.775216	0.794791
O	0.896928	0.724783	0.294791
O	0.103072	0.275217	0.705209
O	0.404666	0.705402	0.307924
O	0.595334	0.294598	0.692076
O	0.595334	0.205401	0.192076
O	0.404666	0.794599	0.807923

NM-M1 (Δ_{V-V} =0.69 Å):

a	5.79684	0	0
b	0	4.429453	0
c	-2.89331	0	4.630266
V	0.233891	0.977386	0.025945
V	0.766109	0.022614	0.974055
V	0.766109	0.477387	0.474055
V	0.233891	0.522613	0.525945
O	0.104176	0.22338	0.204104
O	0.895824	0.77662	0.795896
O	0.895824	0.72338	0.295896
O	0.104176	0.27662	0.704104
O	0.404929	0.704234	0.306407
O	0.595071	0.295766	0.693593
O	0.595071	0.204233	0.193593
O	0.404929	0.795767	0.806407

NM-M1 (Δ_{V-V} =0.55 Å):

a	5.79684	0	0
b	0	4.423595	0
c	-2.89331	0	4.630266
V	0.23710	0.97992	0.02096
V	0.76291	0.02008	0.97904
V	0.76291	0.47992	0.47904
V	0.23710	0.52008	0.52096
O	0.10451	0.22300	0.20210
O	0.89549	0.77700	0.79790
O	0.89549	0.72300	0.29790
O	0.10451	0.27700	0.70210
O	0.40575	0.70460	0.30677
O	0.59425	0.29540	0.69323
O	0.59425	0.20460	0.19323
O	0.40575	0.79540	0.80677

NM-M1 (Δ_{V-V} =0.42 Å):

a	5.79684	0	0
b	0	4.419385	0
c	-2.89331	0	4.630266
V	0.240299	0.983175	0.015969
V	0.759701	0.016825	0.984031
V	0.759701	0.483175	0.484031
V	0.240299	0.516825	0.515969
O	0.104804	0.222344	0.200784
O	0.895196	0.777656	0.799216
O	0.895195	0.722344	0.299216
O	0.104805	0.277656	0.700784
O	0.406371	0.704325	0.306812
O	0.593629	0.295675	0.693188
O	0.593629	0.204325	0.193188
O	0.406371	0.795675	0.806812

FM-M1 (low):

a	5.79684	0	0
b	0	4.482909	0
c	-2.875914	0	4.602425
V	0.24567	0.99424	0.01203
V	0.75433	0.00576	0.98797
V	0.75433	0.49424	0.48797
V	0.24567	0.50576	0.51203
O	0.10030	0.20900	0.20360
O	0.89970	0.79100	0.79640
O	0.89970	0.70900	0.29640
O	0.10030	0.29100	0.70360
O	0.40157	0.69828	0.30660
O	0.59843	0.30172	0.69340
O	0.59843	0.19828	0.19340
O	0.40157	0.80172	0.80660

References

1. Marini, C. *et al*. Optical properties of $V_{1-x}Cr_xO_2$ compounds under high pressure. *Phys. Rev. B* **77**, 235111 (2008).

2. Kim, H. *et al*. Raman study of electric-field-induced first-order metal-insulator transition in VO_2-based devices. *Appl. Phys. Lett.* **86**, 242101 (2005).

3. Arcangeletti, E. *et al*. Evidence of a pressure-induced metallization process in monoclinic VO_2. *Phys. Rev. Lett.* **98**, 196406 (2007).

4. Fujimori, A., Bocquet, A., Saitoh, T. & Mizokawa, T. Electronic structure of 3d transition metal compounds: systematic chemical trends and multiplet effects. *J. Electron Spectrosc.* **62**, 141-152 (1993).

5. Tokura, Y. & Nagaosa, N. Orbital physics in transition-metal oxides. *Science* **288**, 462-468 (2000).

6. Goodenough, J. B. The two components of the crystallographic transition in VO_2. *J. Solid State Chem.* **3**, 490-500 (1971).

7. Hearn, C. Phonon softening and the metal-insulator transition in VO_2. *J. Phys. C: Solid State Phys.* **5**, 1317 (1972).

8. Aetukuri, N. B. *et al*. Control of the metal-insulator transition in vanadium dioxide by modifying orbital occupancy. *Nat. Phys.* **9**, 661 (2013).

9. Zylbersztejn, A. & Mott, N. F. Metal-insulator transition in vanadium dioxide. *Phys. Rev. B.* **11**, 4383 (1975).

10. Dankwort T, Strobel J, Chluba C, et al. Martensite adaption through epitaxial nano transition layers in TiNiCu shape memory alloys. J. Appl. Crystallogr. **49**(3), 1009-1015 (2016).
